# Chromothripsis is a frequent event and underlies typical genetic changes in early T-cell precursor lymphoblastic leukemia in adults

**DOI:** 10.1038/s41375-022-01671-5

**Published:** 2022-08-16

**Authors:** Silvia Arniani, Valentina Pierini, Fabrizia Pellanera, Caterina Matteucci, Danika Di Giacomo, Valentina Bardelli, Martina Quintini, Elena Mavridou, Anair Graciela Lema Fernandez, Carlotta Nardelli, Martina Moretti, Paolo Gorello, Barbara Crescenzi, Silvia Romoli, Donatella Beacci, Marco Cerrano, Nicola Fracchiolla, Simona Sica, Fabio Forghieri, Fabio Giglio, Michela Dargenio, Loredana Elia, Roberta La Starza, Cristina Mecucci

**Affiliations:** 1 Department of Medicine and Surgery, Center for Hemato-Oncology Research (CREO), Hematology and Bone Marrow Transplantation Unit, Department of Medicine and Surgery, University of Perugia, Perugia, Italy; 2 Department of Chemistry, Biology and Biotechnology, University of Perugia, Perugia, Italy; 3 MC: Division of Hematology, Department of Oncology, A.O.U. Città della Salute e della Scienza di Torino, Torino, Italy; 4 UOC Ematologia, Fondazione IRCCS Ca’ Granda-Ospedale Maggiore Policlinico, Milan, Italy; 5 Department of Diagnosis, Oncologic and Hematologic Radiotherapy, Fondazione Policlinico Universitario A. Gemelli IRCCS, Rome, Italy; 6 Hematology Division, Department of Oncology and Hematology, A.O.U of Modena-Policlinico, Modena, Italy; 7 Haematology and Bone Marrow Transplant Unit, IRCCS San Raffaele Scientific Institute, Milan, Italy; 8 Hematology, Department of Translational and Precision Medicine, S.C. Ematologia, Ospedale Vito Fazzi, Lecce, Italy; 9 Division of Hematology, Department of Translational and Precision Medicine, Sapienza University of Rome, Rome, Italy

**Keywords:** Leukaemia, Acute lymphocytic leukaemia

## Abstract

Chromothripsis is a mitotic catastrophe that arises from multiple double strand breaks and incorrect re-joining of one or a few chromosomes. We report on incidence, distribution, and features of chromothriptic events in T-cell acute lymphoblastic leukemias (T-ALL). SNP array was performed in 103 T-ALL (39 ETP/near ETP, 59 non-ETP, and 5 with unknown stage of differentiation), including 38 children and 65 adults. Chromothripsis was detected in 11.6% of all T-ALL and occurred only in adult cases with an immature phenotype (12/39 cases; 30%). It affected 1 to 4 chromosomes, and recurrently involved chromosomes 1, 6, 7, and 17. Abnormalities of genes typically associated with T-ALL were found at breakpoints of chromothripsis. In addition, it gave rise to new/rare alterations, such as, the *SFPQ::ZFP36L2* fusion, reported in pediatric T-ALL, deletions of putative suppressors, such as *IKZF2* and *CSMD1*, and amplification of the *B*C*L2* gene. Compared to negative cases, chromothripsis positive T-ALL had a significantly higher level of *MYCN* expression, and a significant downregulation of *RGCC*, which is typically induced by *TP53* in response to DNA damage. Furthermore we identified mutations and/or deletions of DNA repair/genome stability genes in all cases, and an association with *NUP214* rearrangements in 33% of cases.

## Introduction

T-cell acute lymphoblastic leukemia (T-ALL), accounts for 15% of pediatric and 20–25% of adult acute lymphoblastic leukemias (ALL). It results from a multistep process leading to the accumulation of multiple genetic abnormalities, that affect cell cycle control, self-renewal capacity, cell differentiation, proliferation and apoptosis, and epigenetic modulation [1].

High expression of (onco)genes, coding for transcription factors pivotal for hematopoiesis and/or T-cell development, have been identified by gene expression profile and shown to distinguish unique T-ALL subgroups, i.e., *TAL/LMO, HOXA, TLX3, TLX1, NKX2–1/2–2*, and *MEF2C* [1]. These oncogenes co-operate with deregulated activity of signaling transduction pathways (*NOTCH*, *JAK/STAT, RAS/MEK/ERK, PI3K/AKT, WNT*), epigenetic modifiers (*EZH2, DNMT3A, PHF6*, *PRC2* members), and/or protein translation (*RPL22, RPL10*) to elicit the leukemia phenotype. Diverse molecular mechanisms, i.e., activating and inactivating mutations, balanced/unbalanced chromosome rearrangements, and/or copy number abnormalities (CNA), underlie gene deregulation [1].

First described by Stephens a et al. [2]. chromothripsis is thought to be a one-step catastrophic event that leads to the generation of multiple rearrangements as a consequence of locally clustered DNA double strand breaks [2–5]. Based on single nucleotide polymorphism array (SNPa), it has been defined by the presence of at least ten changes in segmental copy number between two or three states [3]. Afterwards, applying whole exome and whole genome sequencing, chromothripsis has been redefined as any rearrangement characterized by at least 3 of the following events: (1) pronounced clustering of breakpoints across the chromosome; (2) copy number profiles oscillating between two/three states; (3) prevalence of regions with interspersed loss and retention of heterozygosity; (4) prevalence of rearrangements affecting a single allele; (5) randomness of DNA fragment joins and order; and (6) ability to “walk” the derivative chromosome by joining breakpoints [4].

The advent of high resolution genome analyses, has greatly enhanced the detection of chromothriptic events which appear to be a widespread phenomenon, with an overall prevalence of ∼50% in different human cancer subtypes, such as lung, esophageal, bladder, prostate, and breast adenocarcinomas [6, 7]. Chromothripsis is considered an early event that promotes cancer development either disrupting tumor suppressor genes or activating oncogenes, through CNA or the generation of fusion genes [7].

In hematopoietic neoplasms, chromothripsis has been found in B-cell acute lymphoblastic leukemia with iAMP21, myelodysplastic syndromes with complex chromosomal aberrations, acute myeloid leukemia, multiple myeloma, and chronic lymphocytic leukemia [8–13]. T-ALL were not included in the analysis of large series of tumors [6, 7, 14]. However, Sanders et al. [15]. showed the occurrence of subclonal chromothriptic events in a case of pediatric T-ALL. In addition, Ratnaparkhe et al. [16]. reported a ∼4% incidence of chromothripsis in sporadic T-ALL. Lastly, germline chromothriptic events have been reported in congenital disorders [17], and are commonly observed in patients with ataxia telangiectasia (A-T), Nijmegen breakage syndrome, and Bloom syndrome, all depending on impaired DNA repair system [17]. Interestingly, these patients also have a high incidence of chromothripsis in solid or hematological tumors, developing in their life [16]. In particular, it appeared to be a frequent phenomenon in T-ALL arising in A-T subjects [16].

From our SNP array study of a large cohort of pediatric and adult T-ALL chromothriptic events emerged in approximately 11% of adult cases, all with an immature phenotype, and provided insight on key underlying biological events.

## Patients

The investigated T-ALL cohort (=103) included 65 adults and 38 children (median age: 24), and had a prevalence of unclassified (38 cases) and immature (39 cases) cases (Supplementary Tables 1–4, and Supplementary methods). They were all characterized by combined interphase fluorescence in situ hybridization assay [18]. *NOTCH1/FBXW7* hot-spot mutations were detected in 60/101 studied. Patients or their parents/guardians gave informed consent for sample collection and molecular analyses, in agreement with the Declaration of Helsinki. The study was approved by the local bio-ethical committee (CER, research project 3397/18).

## Materials and methods

Karyotyping was done after G-banding with Wright stain and described according to the ISCN (2021). Single nucleotide polymorphism array (SNPa), RNA microarray analysis, Sanger and targeted sequencing are fully described in Supplementary methods (see also Supplementary Tables 1–4 for patient characteristics). Details on RNA sequencing and Whole Genome Sequencing (WGS), performed by Novogene (Cambridge, UK), and data analysis are provided in Supplementary methods.

## Results

### SNP array and WGS

Overall, SNPa detected 830 events (Supplementary Table 5). There were 536 losses, 225 gains, and 69 copy neutral loss of heterozygosity (cnLOH). A significantly higher number of events were found in ETP/near ETP (media/median of 13/11 per case, range: 0–79) than in non-ETP cases (media/median 5/5, range: 0–14) (unpaired *t* test: *p* value 0.0003). Furthermore, an unequal distribution of copy number abnormalities (CNA) was observed among the main genetic subgroups: cases of the *TAL/LMO* subgroup had the lowest number of events (median 3; range 1–12) while cases belonging to the *TLX1* subgroup the highest (median 8.5; range 4–12) (unpaired *t* test: *p* value 0.0087) (Supplementary Table 5).

### Chromothriptic patterns

According to published criteria [3, 4], chromothriptic events were detected in 12/103 patients (11.6%), all with an immature phenotype (10 ETP and 2 near-ETP) (Table 1). They had a range of 10–23 segmental CNA in a 50–218 Mb region, and involved one (=7 cases), two (=3 cases), three (=1 case), or four (=1 case) chromosomes. Chromosomes 1, 6, 7, and 17, were recurrently affected (Table 1). All chromothriptic events were confirmed by WGS in the 5 cases analyzed (Supplementary Table 6).

**Table 1:** Clinical, hematological, and molecular-cytogenetic features of 12 T-ALL cases with chromothripsis.

Case	S/A	Karyotype	*NOTCH1/FBXW7*	Primary event	Additional events	Chromothriptic events	Epigenetic alterations
1	F/27	n.a.	*NOTCH1* c.5161 G > A p.V1721M c.7470 C > A p.Y2490*	*SET-NUP214*	*TCF7* del *SEC63* del *WT1* del *ATM* del *TP53* del *RB1* del	arr(5q)cth	
2	M/19	46,XY[15]	*NOTCH1* c.7488_7496delCACCCCCAGinsGGTTAGTGCCCACAA p.N2496_S2499delinsKVSAHN	*SET-NUP214*	*CASP8AP2-GRIK2-SEC63* del *WT1* del *ETV6-CDKN1B* del *RB1* del *TP53* del *NF1-SUZ12* del	arr(1,17)cth	*SUZ12* del
3	M/30	46,XY,del(4)(q21q28),del(5)(q14q34),del(11)(p15p12),del(12)(p13p12)[3]	wild type	*SET-NUP214*	*TCF7* del *WT1* del *ETV6-CDKN1B* del *NF1-SUZ12* del	arr(16)cth	*EZH2* mut *SUZ12* del
4	M/20	46,XY,?del(6)(p21p25)[15]	wild type	*SQSTM1-NUP214*	Terminal 5q del *CDKN2AB* del *WT1* del *NF1-SUZ12* del	arr(13q)cth	*SUZ12* del
5	M/32	n.a.	*NOTCH1* c.4799 T > A p.L1600Q c.6963–6966dupCCAA p.Y2323Pfs*32	*TRB-HOXA*	*TCF7* del *CASP8AP2* del *CDKN2AB* del trisomy 8	arr(6,7,9)cth	
6	F/19	45–47,XX,add(1)(p36),add(3)(p24),**r(6)(?)**,inv(7)(p15q34),+20,+21[cp8] 46,XX[1]	*FBXW7* c.1322 G > A (p.R441Q)	*TRB-HOXA*	*GRIK2-SEC63-FYN* del *MYB* gain *PIM1* translocation *CDKN2AB* del trisomy 20 trisomy 21	arr(6,8)cth	*EZH2* mut
7	M/34	47–48,XY, + Y,dup(1)(p31p35),**-7**,+mar1,+mar2,+ring[cp10]	*NOTCH1* c.4746delGinsCGCT p.Pro1582_Glu1583insAla c.7328_7329insCT p.Gln2444Cysfs*34	*TRB-HOXA*	*LCK-TAL1-SIL-JAK1* gain	arr(7)cth	*EZH2* mut
8	M/22	46,XY[16]	wild type	*Mir181-HOXA*	*TCF7* del *ETV6-CDKN1B* del *RB1-DLEU1-DLEU7* del *TP53* del *NF1-SUZ12* del	arr(12,17,18,20)cth	*SUZ12* del
9	M/37	n.a.	wild type	*PICALM-MLLT10*	*TCF7* del *CASP8AP2-GRIK2* del *MYC* gain *CDKN2AB* del *ATM* del	arr(2,6)cth	
10	M/24	n.a.	wild type	Undetermined	*GRIK2-SEC63-FYN* del *TRB* del *CDKN2AB* del *ETV6-CDKN1B* del	arr(7)cth	
11	M/23	46,XY,**t(1;2)(p34;p21)**,add(5p),add(Xp),-7,+mar[8] 46,XY[2]	*NOTCH1* c.7017insT p.A2339fs*15	Undetermined	*IKZF1* del *ETV6-CDKN1B* del *RB1-DLEU1-DLEU7* del *NF1-SUZ12* del	arr(1p)cth	*ASXL1* mut *SUZ12* del
12	F/62	n.a.	*NOTCH1* c.4740insTGGCCG p.M1580insWP	undetermined	*TCF7* del *CASP8AP2-GRIK2-SEC63-FYN* del *ETV6-CDKN1B* del *RB1-D13S319* del *NF1-SUZ12* del	arr(1p)cth	*DNMT3A* mut *SUZ12* del

*S* sex, *A* age, *F* female, *M* male, *n.a.* not available, *del* deletion, *mut* mutation. Undetermined means that Fluorescence in situ hybridization did not detect any known primary change; the column “additional events” reports molecular-cytogenetic abnormalities detected by CI-FISH on interphase nuclei (see ref. ^18^).

As expected, cth+ cases had a significantly higher number of events (media/median 29.3/26) than chromothripsis-negative (cth−) ones, the latter including both ETP/near-ETP (media/median 5/5) and non-ETP cases (media/median 5/4) (unpaired *t* test: *p* value 0.0001) (Supplementary Table 7). Instead, among cth− cases, the incidence of CNA and cnLOH did not differ between ETP/near-ETP and non-ETP ALL cases (Supplementary Table 7).

### Chromothripsis generated T-ALL-related oncogenic events

Overall, 14 genes typically involved in T-ALL were affected in chromothriptic rearrangements (Supplementary Tables 8, 9). However, they also affected genes/regions not yet related to human cancer, or known to be involved in solid tumors, or leukemia subtypes other than T-ALL.

#### Primary oncogenic abnormalities

**Fig. 1:**
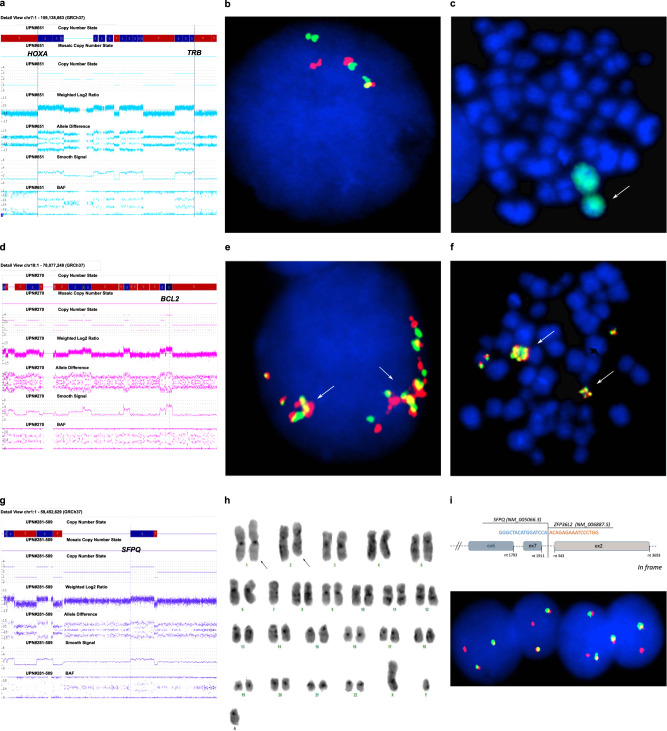
Known and novel oncogenic events associated with chromothripsis. Chromothripsis generated oncogenetic events in T-ALL: **a** SNPa profile of chromosome 7 showing breakpoints within the *HOXA* gene cluster and *TRB@* (case no. 7, Table 1); **b** A double color double fusion FISH assay confirmed the *TRB@::HOXA* rearrangement in an interphase nucleus, i.e., 2 fusions, 1 orange and 1 green signal (case no. 7); **c** Metaphase FISH, with whole chromosome paint 7 (green), showed that one chromosome 7 was involved in a ring chromosome (white arrow) (case no. 7); **d** SNPa profile of chromosome 18 with a gain at *BCL2* locus (case no. 8, Table 1); **e**, **f** FISH with the LSI *BCL2* break-apart FISH probe (Vysis-Abbott, Milan, Italy) confirmed the gene was present as clusters of multiple fusion signals in interphase nuclei (**e**) and in an abnormal metaphase (**f**) (white arrows); **g** SNPa profile of chromosome 1 with a breakpoint at *SFPQ* (case no. 11, Table 1); **h** The G-banded karyotype of case no. 11 showing the reciprocal translocation between chromosomes 1 and 2 (black arrows); **i** The *SFPQ-ZFP36L2 in frame* fusion transcript detected by RNA sequencing (upper part); the rearrangement of *ZFP36L2* was confirmed by the break-apart FISH assay with RP11–339H12 (orange) and RP11–391M15 (green) that gave 2 fusions and 1 orange signal) indicating a breakpoint occurred within clone RP11–339H12 (case no. 11).

The typical *TRB@::HOXA* rearrangement was produced by chromothripsis in two cases (nos. 5 and 7, Table 1). In both, chromosome 7 had segmental copy number changes, at 7p15 band, within the *HOX*A cluster, and at 7q34, within the *TRB@* locus (Fig. 1a). FISH confirmed the *TRB@::HOXA* rearrangement in both (Fig. 1b), and demonstrated the structural involvement of #7 in a ring chromosome (Fig. 1c), in case no. 7.

#### Genomic losses typically associated with T-ALL

A least 13 oncosuppressor genes which are recurrently deleted in T-ALL, were lost due to chromothripsis (Supplementary Table 9 and Supplementary Fig. 1). In three cases (nos. 2, 11, and 12), chromothripsis at chromosome 1p produced a deletion with a shared 470 kb region, at 1p36.32-p36.31. This region, which was also deleted in five cth− T-ALL cases, encompasses two putative oncosuppressor genes, i.e., *CHD5* and *RPL22*. At chromosome 5, it caused the deletion of *TCF7*, at 5q31.1 (case no. 1). In the 3 cases with chromothripsis at the long arm of chromosome 6 (nos. 5, 6, and 9), a common deleted region (CDR) of 2,7 Mb, at 6q15, involved the lncRNA *TSG1*, and the ephrin receptor *EPHA7*. This CDR was also found in 14 cth− cases, with variable interstitial del(6q) (Supplementary Table 9). Among cases with a chromotriptic chromosome 7, case no. 10 had a 2.9 Mb deletion, at 7p12.3-p12.1, involving *IKZF1*, which was also deleted in 4 cth− T-ALL, whereas case no. 3, had a deletion of *EZH2*, at 7q36. At chromosome 9, the deletion of *CDKN2AB* was produced by chromothripsis in case no. 5. A chromothriptic event at chromosome 12p13 provoked the deletion of both *ETV6* and *CDKN1B* (case no. 8). In case no. 3, with a single chromothriptic chromosome 16, the oncosuppressor gene *CTCF* underwent partial monoallelic loss; *CTCF* was also deleted in 9 cth− cases. Likewise, chromothripsis of chromosome 17, caused loss of *TP53/*17p13.1 and of *SUZ12/*17q11 in 2 cases (nos. 2 and 8) (all patient numbers referred to Table 1). The overall number of T-ALL cases harboring these deletions, is indicated in Supplementary Table 9.

#### New CNAs and fusion transcripts

Chromothripsis caused focal CNA at new putative oncogenic regions/genes (Supplementary Table 9), as observed in case no. 9, with chromothripsis at chromosome 2 and whole or partial deletion monoallelic of *NUP35, TFPI, CREB1* and *IKZF2*; in case no. 6 with chromothripsis at chromosome 8, and a focal deletion of the oncosuppressor gene *CSMD1* [19]; and in case no. 8, in which chromothripsis produced the amplification of *BCL2*, at 18q21.33 (copy number state > 4) (Fig. 1d–f) (patient numbers referred to Table 1). The 3’*CSMD1* partial deletion and *BCL2* amplification were confirmed by FISH (Supplementary Results).

Besides CNA, the aberrant recombination of genes at chromothripsis breakpoints generated fusion transcripts, as demonstrated by RNASeq studies (Supplementary results and Supplementary Table 10). In case no. 1 (Table 1), chromothripsis at chromosome 5 disrupted *STARD4*/5q22.1, with loss of the 3’region, and *TCERG1*/5q32, that lost the 5’ end, and produced the *STARD4::TCERG1* fusion; FISH confirmed the 5q22.1-q32 interstitial deletion and the gene fusion (Supplementary Results). In case no. 11 (Table 1), chromothripsis at chromosome 1p (Fig. 1g) was associated with a reciprocal t(1;2)(p36;p21) translocation (Fig. 1h) which involved the splicing factor *SFPQ* (1p34.3) and *ZFP36L2* (2p21), and produced the *SFPQ::ZFP36L2* fusion (Fig. 1i, upper panel). The rearrangement of *ZFP36L2* was also detected by FISH, using a specific dual-color break-apart assay, in diagnostic and relapse samples, but not in hematological remission (Fig. 1i, lower panel) (Supplementary results). Supervised hierarchical analysis showed that this case clustered within the *HOXA* subgroup (Supplementary Fig. 3) and had high levels of *HOXA13* expression (Supplementary Fig. 4).

### Differentially expressed genes in cth+ immature T-ALL

**Fig. 2:**
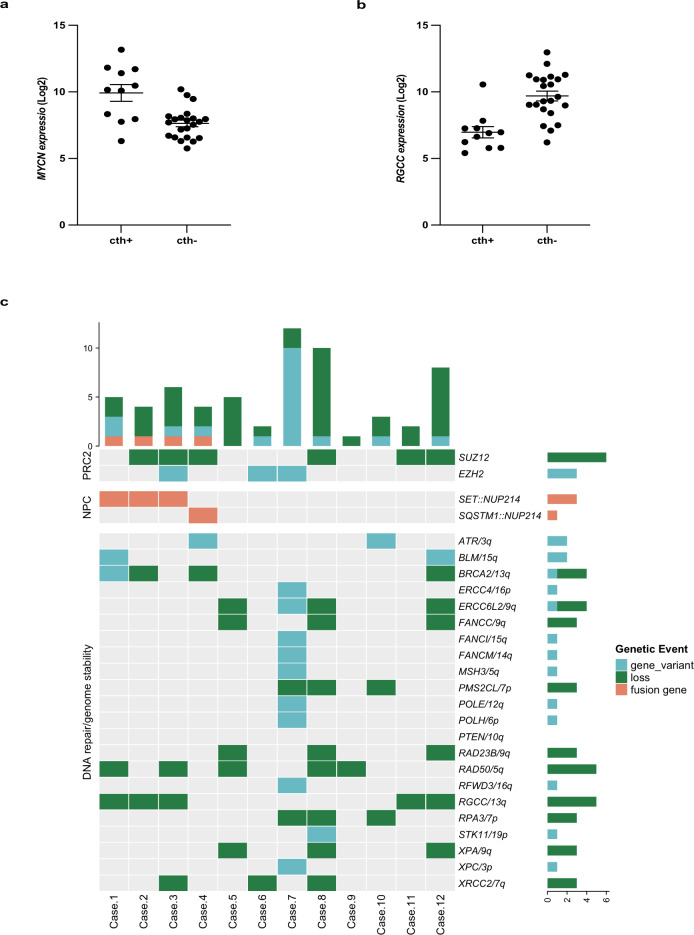
Genetic events associated with chromosome instability in T-ALL with chromothripsis. RNA microarray showed that: **a** the levels of *MYCN* expression were significantly higher in chromothripsis positive (cth+) (=11 cases) than chromothripsis negative (cth−) (=22 cases) cases (FDR *p* value 0,0215) (Mann–Whitney test, *p* = 0.0002) and that; **b** *RGCC* expression was significantly lower in cth+ (=11) than cth− (=22) cases (FDR *p* value 0,0254) (Mann–Whitney test, *p* < 0.0001). The expression of *MYCN* and *RGCC* was indicated as log signal. **c** The heat-map reports on the types and distribution of alterations affecting DNA repair/chromosome stability genes, in the 12 cth+ T-ALL cases (patient numbers refer to Table 1). Bar graphs, i.e., top left and bottom right, respectively indicate the number of genomic events for each case and the number of events for each gene. Loss: monoallelic deletion.

Supervised hierarchical RNA microarray analysis of cth+ (=11) *vs* cth− (=57) cases identified 121 differentially expressed genes (DEG): 65 down- and 56 up- regulated (fold change ≥ ±2.0 and FDR < 0.05) (Supplementary Table 11). Cth+ cases were characterized by a significant over-expression of *MYCN*, *GATA2* and *CD33*, as observed in immature T-ALL, and down-regulation of *ATM* and *RGCC*, both involved in DNA Double-Strand Break Repair and Apoptosis; no other DNA repair/mismatch or genomic stability genes belonged to the DEGs. Focusing on immature T-ALL (=33), RNA microarray revealed that 64 DEG (44 down- and 20 up- regulated) distinguished cth+ (=11) from cth− (=22) cases: *MYCN* (up-regulated; fold change 5.57) (Fig. 2a) and *RGCC* (down-regulated; fold change of −8.02) were amongst the top deregulated genes (Supplementary Table 13 and, Fig. 2b).

RNA-Seq identified 111 DEGs, i.e., 50 down- and 61 up-regulated, that distinguished cth+ from cth− T-ALL cases (Supplementary Fig. 2a, Supplementary Table 12), and confirmed the downregulation of *RGCC*. Functional analysis showed the downregulation of nitric oxide related genes (i.e., *AOX1* and *HBA1*) and the upregulation of the *NOTCH* signaling (including *MFAP2*, *JAG2* and *DLK1* genes), as the top enriched pathways in cth+ T-ALL (FDR < 0.1) (Supplementary Fig. 2b). Moreover, the expression profile of cth+ cases was characterized by the over-expression of *EZH2* targets (FDR < 0.1) (Supplementary Fig. 2c). Specifically, 30 out of 111 DEGs (27%) were *EZH2* targets and 24 of them were upregulated (Supplementary Table 12).

### Recurrent genomic abnormalities in cth+ T-ALL

Conventional cytogenetics was informative in 7/12 cth+ T-ALL cases (Table 1). The karyotype was complex (≥3 abnormalities) in 4 cases, it showed 1 structural abnormality in 1, while it was normal in 2. CI-FISH detected *HOXA cis−* (*TRB::HOXA* in 3, *mir181::HOXA* in 1) or *trans*- activating abnormalities (*SET*::*NUP214* in 3, *SQSTM1::NUP214* in 1, *PICALM::MLLT10* in 1), in 9 cases (Table 1), while the primary oncogenic abnormality was not identified in the remaining 3. Recurrent monoallelic deletions affected *TP53* (=3), *RB1* (=5), *TCF7* (=6), *CDKN1B* (=6), and the 6q14-q15 region (=7) (Table 1). *NOTCH1/FBXW7* hot-spot mutations were found in 7/12 cases, while no pathogenetic variants of *TERT* promoter, or *TERT* rearrangements/CNA were identified. Likewise, no abnormalities of *MYCN* were detected.

The Sophia Myeloid Solution (Sophia Genetics) detected 18 gene variants (range: 1–4 per case), in 10/12 cth+ cases (Supplementary Table 14), with a variant allele fraction (VAF) ranging from 32% to 96,6%. Recurrent mutations of transcription factors, i.e., *WT1*, *ETV6*, and *RUNX1* (2 cases each), as well as of epigenetic modulators, i.e. *IDH2* (=1), *ASXL1* (=1), *DNMT3A* (=1), and *EZH2* (=3) were identified. Overall, targeted sequencing and SNPa detected 1–2 mutations/deletions of epigenetic modulators in 8 cases (Table 1) (Fig. 2c).

### DNA/mismatch repair and genomic stability genes

Among CNA identified by SNPa, monoallelic losses encompassing *BRCA2, ERCC6L2, FANCC, PMS2CL, RAD23B, RAD50, RGCC, RPA3, XPA* and *XRCC2*, were significantly associated with cth+ T-ALL cases. As reported above, only *RGCC* was significantly downregulated (Supplementary Table 15).

Overall, the Hereditary Cancer Solution (Sophia Genetics) detected 16 single nucleotide variants (SNV) (9 in cth+ and 7 in cth− T-ALL cases) with a VAF range from 10.1% to 51.8% (Supplementary Table 16). Among them, 4 somatic pathogenic/likely pathogenic variants were identified in three cases of cth+ T-ALL: *BRCA2* (=1), *STK11* (=1), and *PTEN* (=2 in a single case). Instead, 3 somatic pathogenic/likely pathogenic variants (1 *ATM*, 1 *MUTYH*, and 1 *PTEN*) were detected in 2 cth− T-ALL cases. Five VUS (4 germline and 1 somatic) were also found (Supplementary Table 16).

RNASeq analysis of 235 candidate genes (Supplementary Table 17), identified two additional pathogenetic variants in 4 cth+ cases. Both variants were previously reported in solid tumors [20]: the *BLM* c.1544del, p.N515Mfs*16 (COSM252959) was found in cases nos. 1 and 12; the *ATR* c.2320del, p.I774Yfs*5 (COSM214499) in cases nos. 4 and 10 (Fig. 2c). WGS analysis of the exonic and splicing regions of the 235 candidate genes (Supplementary Table 17), detected pathogenetic/likely pathogenetic somatic SNV and/or insertion/deletion (Indel), in all cases. Specifically, cases UPN878, UPN880 and UPN884 had 3 variants, UPN886 5, and UPN882 20 (Supplementary Tables 18, 19). Overall, copy number abnormalities and/or variants of DNA repair/genomic stability genes were detected in all cases (range of events: 1–9; median: 3) (Fig. 2c) (Supplementary Table 19).

### Clinical characteristics of patients with cth+ T-ALL

Chromotripsis was only detected in the adult T-ALL population, predominantly in young patients (age range: 19–62; median = 25.5 years) (Table 1). There were 8 males and 4 females. All cases displayed an immature phenotype and were classified as ETP (=10) (cases nos. 1, 3–7, 9–12, Table 1) or near-ETP ALL (=2) (cases nos. 2 and 8, Table 1) [1, 18, 21–23]. The follow-up was available in all patients: 3 of them were treated with pediatric-oriented protocols and were alive at 30 (no. 6, AIEOP-BFM ALL 2009, NCT01117441), 10 (case no. 3) and 48 (case no. 8) months from diagnosis (the latter two cases were treated according to the GIMEMA LAL1913-NCT02067143 induction phase and after achieving the complete remission underwent unrelated hematopoietic stem cell transplantation); 9 patients died from disease progression/relapse 1–48 months after diagnosis (median: 12 months) (Supplementary Table 20).

## Discussion

Chromothripsis is a widespread phenomenon in human cancers, reported in both solid and hematological tumors [6, 7], and linked with poor outcome [7, 8]. So far, no studies are available on its incidence, distribution, and features in sporadic T-ALL.

By applying a whole genome SNP array to a wide range of pediatric and adult T-ALL, we estimated that chromothripsis occurred in ~11% of T-ALL, specifically in 30% of ETP/near-ETP of Adults. Correlation with prognosis and outcome was precluded due to the relatively small cohort of patients and heterogenous treatments.

The genomic profile of chr+ cases was largely overlapping with that of chr- ETP/near ETP ALL, as both subgroups showed a high rate of abnormalities associated with *HOXA* over-expression and recurrent deletions of *TCF7, CDKN1B, RB1*, and/or *NF1*. In addition, abnormalities of epigenetic modulators, typical for immature T-ALL, were detected in 66% of cth+ cases [1, 18].

We found that cytogenetics did not reflect the genomic instability of the disease, as chromothripsis was indifferently present in cases with normal and complex karyotypes. Although in T-ALL of patients with Ataxia Telangiectasia the typical pattern of chromothripsis involved acrocentric chromosomes [16], this group was not specifically affected in sporadic T-ALL. In fact, we found the recurrent involvement of chromosomes 1, 6, 7, and 17. Similar results were found by Zhang et al. [24], who detected chromothripsis on metacentric and submetacentric chromosomes but not on acrocentric ones, in ETP-ALL. Moreover, hotspot regions leading to specific copy number changes and breakpoints were identified.

### Chromothripsis is an oncogenetic mechanism

As regards the specific hotspots, chromothripsis must be counted among the molecular mechanisms that generate oncogenic events typical of T-ALL, similarly to what has been observed in other human tumors. Interestingly, besides producing rearrangements and CNA of oncogenes/oncosuppressors recurrently involved in T-ALL, such as *RPL22*, *TCF7*, *IKZF1*, *EZH2*, *CDKN2AB, ETV6*, *CDKN1B*, *TP53*, and rearrangement of the *HOXA* locus, our study provided evidence that it also generated new putative leukemogenic events, broadening the spectrum of oncogenic lesions linked to T-ALL pathogenesis/progression. Although detected in single cases, some of these events deserve attention. For istance, at chromosome 2q34, chromothripsis caused loss of the transcription factor *IKZF2* which frequently undergoes intragenic deletions in adult T-cell leukemia/lymphoma (ATL) [25], and that we also found deleted in ∼3% of our cth− T-ALL cases. Similarly, deletion of *CSMD1* (CUB and Sushi multiple domains 1), a tumor suppressor gene at 8p23, which has been associated with poor prognosis in many solid tumors, including breast, gastric, squamous, head and neck, and hepatocellular carcinomas [19, 26] emerged as recurrent CNA in T-ALL, occurring in 2% of cth− cases. In a single case with chromothripsis at 18q, we observed the amplification of *BCL2*, that fits with the well-established upregulation of *BCL2* in immature T-ALL [24].

Chromothripsis also produced the *SFPQ::ZFP36L2* fusion in a case assigned to the *HOXA* subgroup of T-ALL, by RNA expression studies. Interestingly, fusions of *ZFP36L2* with different partners, including *SFPQ*, have been reported in pediatric and adult T-ALL without evidence of subtype-specific oncogenic rearrangements, as in our case [22, 27].

*ZFP36L2* (zinc finger protein 36-like 2) is a critical factor in hematopoiesis, where it has a role in maintaining self-renewal of early colony forming erythroid progenitors [24, 28]. In thymocyte development it is expressed at high levels in CD4+ T cells [29, 30]. Targets of *ZFP36L2* include oncogenes and tumor suppressors, such as *NOTCH1*, *EZH2, MYC, SPI.1, LMO2, LYL1*, and *TAL1*. Interestingly, *ZFP36L2* knock-out mice develop T-ALL [29]. Overall, these data indicate the putative oncogenic role of the *SFPQ::ZFP36L2* fusion in T-ALL, especially related to the *HOXA* subgroup.

### Chromothripsis and chromosome instability

Our study identified three main factors involved in chromosome instability in cht+ T-ALL, in particular, upregulation of *MYCN*, defects of DNA repair/genome stability genes, and genetic abnormalities at *NUP214* nuclear pore complex (NPC) gene.

An association between complex genomic rearrangements/catastrophic events and DNA repair defects, inefficient apoptosis, and *MYC/MYCN* amplification/overexpression has been reported in mouse models [14]. Likewise, mutations of DNA repair genes, *TP53* inactivation, and *MYC*/*MYCN* amplification/overexpression, co-occur in diverse human tumors harboring chromothriptic events [14].

RNA microarrays showed that *MYCN* was one of the three most up-regulated genes distinguishing cth+ from cth− ETP/near-ETP ALL cases. *MYCN*, a negative regulator of *NDRG*1 (N-myc downstream-regulated gene 1), has a pivotal role in spindle checkpoint, microtubule functioning, and centrosome homeostasis [31–33]. The role of *MYCN* in controlling genome stability has been strengthened in transgenic zebrafish, where, mismatch repair, homologous recombination, and base excision repair pathways are typically abolished [33]. Our findings not only confirmed that high levels of *MYCN* are typically associated with an immature phenotype [34] but also provided the first evidence of an association between *MYCN* expression and the occurrence of chromothripsis in human immature T-ALL.

Elevated levels of *MYCN* have been related to both gain/amplification and the L44P hot-spot variant [14, 22, 35] which were not found in our series. Instead, alterations of the *PRC2* complex, one of the major negative regulators of *MYCN*, were present in 66% of our cases which harbored *SUZ12* deletions and/or *EZH2* mutations. The abrogation of the *PRC2* activity may explain the high levels of *MYCN* expression in cth+ ETP-ALL [36] as observed in T-ALL cell lines, where induced *EZH2* inactivation correlates with an immature signature, transcriptional up-regulation of *MYCN*, and an increased *MYCN*-driven replication stress [37]. In agreement with these findings, the signature of our cth+ ETP-ALL was characterized by deregulation of as many as 30 *EZH2* targets.

As reported in solid tumors [14], *MYCN* overexpression was associated with abnormalities of DNA repair/genome stability genes in all our cth+ T-ALL cases, consistent with the hypothesis that DNA repair factors play a central role in the onset of chromothripsis [7, 14]. Loss of at least one DNA repair gene was found in all of our cth+ cases, while non-silent variants of *ATR, BLM, BRCA2, MSH3, ERCC4, ERCC6L2, FANCI, FANCM, POLE, POLH, PTEN, RFWD3, STK11*, and/or *XPC* were detected in 7/12 cth+ cases, with *ATR* and *BLM* as the only recurrently involved genes. In contrast, variants of *ATM* and/or *TP53*, which are closely linked to chromothripsis in human cancers, were not found [6–8]. Interestingly, deletion/down-regulation of *RGCC*, encoding for a protein which is induced by p53 in response to DNA damage [38, 39] was significantly associated with cth+ T-ALL. *RGCC* behaves as putative oncosuppressor in pediatric B-ALL, multiple myeloma, and other tumor types, where it was found to be epigenetically silenced [40, 41].

Lastly, alterations of *NUP214*, a component of the nuclear pore complex (NPC), which is known to play a pivotal role in preserving the genome integrity, were detected in 33% of cases [42, 43]. It is worth mentioning, that NUP214 binds and stabilizes the DDX19 RNA helicase, a protein that contributes to maintain the genomic stability, by preventing spontaneous DNA damage [44]. This function is likely abrogated in cells with *SET::NUP214* or *SQSTM1::NUP214*, as both fusion proteins localize in specific nuclear bodies and do not exert their shuttling activity [45].

In conclusion, this study provided new insights to the understanding of the genomic complexity of immature T-ALL. In particular, chromothripsis emerged as a frequent mechanism generating major oncogenetic events in a subset of ETP/near-ETP of young adults. A wider case series needs to be studied to estimate whether chromothripsis has a negative clinical impact in T-ALL, as in solid tumors.

## Supplementary information

Supplementary Information

Supplementary Tables (final)

## Data Availability

Data were deposited at the public repository Gene Expression Omnibus (GEO) (http://www.ncbi.nlm.nih.gov/geo/) with accession number GSE205270.

## References

[1] Bardelli V, Arniani S, Pierini V, Di Giacomo D, Pierini T, Gorello P, et al. T-cell acute lymphoblastic leukemia: Biomarkers and their clinical usefulness. Genes (Basel). 2021;12. 10.3390/genes12081118. 10.3390/genes12081118 PMC8394887 34440292

[2] Stephens PJ, Greenman CD, Fu B, Yang F, Bignell GR, Mudie LJ (2011). Massive genomic rearrangement acquired in a single catastrophic event during cancer development. Cell.

[3] Rausch T, Jones DTW, Zapatka M, Stütz AM, Zichner T, Weischenfeldt J (2012). Genome sequencing of pediatric medulloblastoma links catastrophic DNA rearrangements with TP53 mutations. Cell.

[4] Korbel JO, Campbell PJ (2013). Criteria for inference of chromothripsis in cancer genomes. Cell.

[5] Rode A, Maass KK, Willmund KV, Lichter P, Ernst A (2016). Chromothripsis in cancer cells: An update. Int J Cancer.

[6] Cortés-Ciriano I, Lee JJK, Xi R, Jain D, Jung YL, Yang L (2020). Comprehensive analysis of chromothripsis in 2,658 human cancers using whole-genome sequencing. Nat Genet.

[7] Voronina N, Wong JKL, Hübschmann D, Hlevnjak M, Uhrig S, Heilig CE et al. The landscape of chromothripsis across adult cancer types. Nat Commun. 2020;11. 10.1038/s41467-020-16134-7. 10.1038/s41467-020-16134-7 PMC7210959 32385320

[8] Fontana MC, Marconi G, Feenstra JDM, Fonzi E, Papayannidis C, Ghelli Luserna Di Rorá A (2018). Chromothripsis in acute myeloid leukemia: Biological features and impact on survival. Leukemia.

[9] Gao J, Chen YH, Mina A, Altman JK, Kim KY, Zhang Y (2020). Unique morphologic and genetic characteristics of acute myeloid leukemia with chromothripsis: a clinicopathologic study from a single institution. Hum Pathol.

[10] Salaverria I, Martín-Garcia D, López C, Clot G, García-Aragonés M, Navarro A (2015). Detection of chromothripsis-like patterns with a custom array platform for chronic lymphocytic leukemia. Genes Chromosom Cancer.

[11] Walker BA, Mavrommatis K, Wardell CP Erratum: Identification of novel mutational drivers reveals oncogene dependencies in multiple myeloma. Blood. 2018;132:587–97. 10.1182/blood-2018-03-840132. 10.1182/blood-2018-03-840132 PMC6097138 29884741

[12] Maura F, Bolli N, Angelopoulos N, Dawson KJ, Leongamornlert D, Martincorena I et al. Genomic landscape and chronological reconstruction of driver events in multiple myeloma. Nat Commun. 2019;10. 10.1038/s41467-019-11680-1. 10.1038/s41467-019-11680-1 PMC6707220 31444325

[13] Maura F, Boyle EM, Rustad EH, Ashby C, Kaminetzky D, Bruno B (2021). Chromothripsis as a pathogenic driver of multiple myeloma. Semin Cell Dev Biol.

[14] Ratnaparkhe M, Wong JKL, Wei PC, Hlevnjak M, Kolb T, Simovic M et al. Defective DNA damage repair leads to frequent catastrophic genomic events in murine and human tumors. Nat Commun. 2018;9. 10.1038/s41467-018-06925-4. 10.1038/s41467-018-06925-4 PMC6232171 30420702

[15] Sanders AD, Meiers S, Ghareghani M, Porubsky D, Jeong H, van Vliet MACC et al. Single-cell analysis of structural variations and complex rearrangements with tri-channel processing. Nat Biotechnol. 2020;38. 10.1038/s41587-019-0366-x. 10.1038/s41587-019-0366-x PMC7612647 31873213

[16] Ratnaparkhe M, Hlevnjak M, Kolb T, Jauch A, Maass KK, Devens F (2017). Genomic profiling of Acute lymphoblastic leukemia in ataxia telangiectasia patients reveals tight link between ATM mutations and chromothripsis. Leukemia.

[17] Nazaryan-Petersen L, Bjerregaard VA, Nielsen FC, Tommerup N, Tümer Z Chromothripsis and DNA repair disorders. J. Clin. Med. 2020;9. 10.3390/jcm9030613. 10.3390/jcm9030613 PMC7141117 32106411

[18] La Starza R, Pierini V, Pierini T, Nofrini V, Matteucci C, Arniani S (2020). Design of a comprehensive fluorescence in situ hybridization assay for genetic classification of T-cell acute lymphoblastic leukemia. J Mol Diagnostics.

[19] Gialeli C, Gungor B, Blom AM (2018). Novel potential inhibitors of complement system and their roles in complement regulation and beyond. Mol Immunol.

[20] Tate JG, Bamford S, Jubb HC, Sondka Z, Beare DM, Bindal N (2019). COSMIC: the catalogue of somatic mutations in cancer. Nucleic Acids Res.

[21] Coustan-Smith E, Mullighan CG, Onciu M, Behm FG, Raimondi SC, Pei D (2009). Early T-cell precursor leukaemia: a subtype of very high-risk acute lymphoblastic leukaemia. Lancet Oncol.

[22] Liu Y, Easton J, Shao Y, Maciaszek J, Wang Z, Wilkinson MR (2017). The genomic landscape of pediatric and young adult T-lineage acute lymphoblastic leukemia. Nat Genet.

[23] Morita K, Jain N, Kantarjian H, Takahashi K, Fang H, Konopleva M (2021). Outcome of T-cell acute lymphoblastic leukemia/lymphoma: Focus on near-ETP phenotype and differential impact of nelarabine. Am J Hematol.

[24] Zhang J, Ding L, Holmfeldt L, Wu G, Heatley SL, Payne-Turner D (2012). The genetic basis of early T-cell precursor acute lymphoblastic leukaemia. Nature.

[25] Kataoka K, Nagata Y, Kitanaka A, Shiraishi Y, Shimamura T, Yasunaga JI (2015). Integrated molecular analysis of adult T cell leukemia/lymphoma. Nat Genet.

[26] Dutta M, Nakagawa H, Kato H, Maejima K, Sasagawa S, Nakano K et al. Whole genome sequencing analysis identifies recurrent structural alterations in esophageal squamous cell carcinoma. PeerJ. 2020;2020. 10.7717/peerj.9294. 10.7717/peerj.9294 PMC7323713 32617189

[27] Chen B, Jiang L, Zhong ML, Li JF, Li BS, Peng LJ (2017). Identification of fusion genes and characterization of transcriptome features in T-cell acute lymphoblastic leukemia. Proc Natl Acad Sci USA.

[28] Stumpo DJ, Broxmeyer HE, Ward T, Cooper S, Hangoc G, Yang JC (2009). Targeted disruption of Zfp36l2, encoding a CCCH tandem zinc finger RNA-binding protein, results in defective hematopoiesis. Blood.

[29] Hodson DJ, Janas ML, Galloway A, Bell SE, Andrews S, Li CM (2010). Deletion of the RNA-binding proteins ZFP36L1 and ZFP36L2 leads to perturbed thymic development and T lymphoblastic leukemia. Nat Immunol.

[30] Makita S, Takatori H, Iwata A, Tanaka S, Furuta S, Ikeda K et al. RNA-binding protein ZFP36L2 downregulates helios expression and suppresses the function of regulatory T cells. Front Immunol. 2020;11. 10.3389/fimmu.2020.01291. 10.3389/fimmu.2020.01291 PMC7324482 32655569

[31] Melotte V, Qu X, Ongenaert M, Criekinge W, Bruïne AP, Baldwin HS (2010). The N‐myc downstream regulated gene (NDRG) family: diverse functions, multiple applications. FASEB J.

[32] Kovacevic Z, Richardson DR (2006). The metastasis suppressor, Ndrg-1: A new ally in the fight against cancer. Carcinogenesis.

[33] Shen LJ, Chen FY, Zhang Y, Cao LF, Kuang Y, Zhong M et al. MYCN Transgenic zebrafish model with the characterization of acute myeloid leukemia and altered hematopoiesis. PLoS One. 2013;8. 10.1371/journal.pone.0059070. 10.1371/journal.pone.0059070 PMC3598662 23554972

[34] Ferrando AA, Neuberg DS, Staunton J, Loh ML, Huard C, Raimondi SC (2002). Gene expression signatures define novel oncogenic pathways in T cell acute lymphoblastic leukemia. Cancer Cell.

[35] Pugh TJ, Morozova O, Attiyeh EF, Asgharzadeh S, Wei JS, Auclair D (2013). The genetic landscape of high-risk neuroblastoma. Nat Genet.

[36] Tsubota S, Kishida S, Shimamura T, Ohira M, Yamashita S, Cao D (2017). PRC2-mediated transcriptomic alterations at the embryonic stage govern tumorigenesis and clinical outcome in MYCN-driven neuroblastoma. Cancer Res.

[37] León TE, Rapoz-D’Silva T, Bertoli C, Rahman S, Magnussen M, Philip B (2020). EZH2 -deficient T-cell acute lymphoblastic leukemia is sensitized to CHK1 inhibition through enhanced replication stress. Cancer Disco.

[38] Saigusa K, Imoto I, Tanikawa C, Aoyagi M, Ohno K, Nakamura Y (2007). RGC32, a novel p53-inducible gene, is located on centrosomes during mitosis and results in G2/M arrest. Oncogene.

[39] Vlaicu SI, Tatomir A, Rus V, Rus H. Role of C5b-9 and RGC-32 in cancer. Front Immunol. 2019;10. 10.3389/fimmu.2019.01054. 10.3389/fimmu.2019.01054 PMC6530392 31156630

[40] Almamun M, Levinson BT, Van Swaay AC, Johnson NT, McKay SD, Arthur GL (2015). Integrated methylome and transcriptome analysis reveals novel regulatory elements in pediatric acute lymphoblastic leukemia. Epigenetics.

[41] Takai N, Kawamata N, Walsh CS, Gery S, Desmond JC, Whittaker S (2005). Discovery of epigenetically masked tumor suppressor genes in endometrial cancer. Mol Cancer Res.

[42] Liu S, Kwon M, Mannino M, Yang N, Renda F, Khodjakov A (2018). Nuclear envelope assembly defects link mitotic errors to chromothripsis. Nature.

[43] Paulsen RD, Soni DV, Wollman R, Hahn AT, Yee MC, Guan A (2009). A genome-wide siRNA screen reveals diverse cellular processes and pathways that mediate genome stability. Mol Cell.

[44] Hodroj D, Recolin B, Serhal K, Martinez S, Tsanov N, Abou Merhi R (2017). An ATR ‐dependent function for the Ddx19 RNA helicase in nuclear R‐loop metabolism. EMBO J.

[45] Port SA, Mendes A, Valkova C, Spillner C, Fahrenkrog B, Kaether C (2016). The oncogenic fusion proteins SET-Nup214 and sequestosome-1 (SQSTM1)-Nup214 form dynamic nuclear bodies and differentially affect nuclear protein and Poly(A)+ RNA export. J Biol Chem.

