# Supplementary material for: Chromothripsis is a frequent event and underlies typical genetic changes in early T-cell precursor lymphoblastic leukemia in adults
Source: Leukemia. 2022 Aug 16;36(11):2577–85. doi: 10.1038/s41375-022-01671-5 (PMC9613476; doi:10.1038/s41375-022-01671-5)

Supplementary Information includes:

Supplementary Materials and Methods

Supplementary Resutls

Supplementary Figures 1, 2, and 3

Chromothripsis is a frequent event and underlies typical genetic changes in early T-cell precursor lymphoblastic leukemia in adults

Silvia Arniani,[1]* Valentina Pierini,[1]* Fabrizia Pellanera,[1]* Caterina Matteucci,[1] Danika Di Giacomo,[1] Valentina Bardelli,[1] Martina Quintini,[1] Elena Mavridou,[1] Anair Graciela Lema Fernandez,[1] Carlotta Nardelli,[1] Martina Moretti,[1] Paolo Gorello,[1,2] Barbara Crescenzi,[1] Silvia Romoli,[1] Donatella Beacci,[1] Marco Cerrano,[3] Nicola Fracchiolla,[4] Simona Sica,[5] Fabio Forghieri,[6] Fabio Giglio,[7] Michela Dargenio,[8] Loredana Elia,[9] Roberta La Starza,[1]° Cristina Mecucci [1]°

[1] Department of Medicine and Surgery, Center for Hemato-Oncology Research (CREO), Hematology and Bone Marrow Transplantation Unit, Department of Medicine and Surgery, University of Perugia, Perugia, Italy

[2] Department of Chemistry, Biology and Biotechnology, University of Perugia, Perugia, Italy

[3] MC: Division of Hematology, Department of Oncology, A.O.U. Città della Salute e della Scienza di Torino, Torino, Italy

[4] UOC Ematologia, Fondazione IRCCS Ca' Granda-Ospedale Maggiore Policlinico, Milan, Italy

[5] Department of Diagnosis, Oncologic and Hematologic Radiotherapy, Fondazione Policlinico Universitario A. Gemelli IRCCS, Rome, Italy

[6] Hematology Division, Department of Oncology and Hematology, A.O.U of Modena-Policlinico, Modena, Italy

[7] Haematology and Bone Marrow Transplant Unit, IRCCS San Raffaele Scientific Institute, Milan, Italy

[8] Hematology, Department of Translational and Precision Medicine, S.C. Ematologia, Ospedale Vito Fazzi, Lecce, Italy
[9] Division of Hematology, Department of Translational and Precision Medicine, Sapienza University of Rome

Supplementary Materials and Methods

Patients. The T-ALL cohort included 103 patients. There were 38 children and 65 adults with a male/female ratio of 2.32 (**Supplementary Table 1**). In 98 cases the immunophenotype (IP) was consistent with a diagnosis of ETP (=33), near ETP (=6),[1–4] and non ETP (=59), while in 5 cases the assignment to a specific subgroup was not possible due to incomplete panel of tested antibodies. Combined interphase fluorescence in situ hybridization assay (CI-FISH)[5] provided the genetic classification of 63 cases (34 *HOXA*, 15 *TAL/LMO*, 8 *TLX3*, and 6 *TLX1*); 2 cases had *SPI.1* fusions, while in the remaining 38 cases abnormalities of known primary oncogenes were not identified. A detailed classification of ETP/near-ETP ALL is provided in **Supplementary Table 2**. Patients or their parents/guardians gave informed consent for sample collection and molecular analyses, in agreement with the Declaration of Helsinki. The study was approved by the local bio-ethical committee (research project 3397/18).

Nucleic acid extraction. Genomic DNA was extracted from bone marrow (BM) or peripheral blood (PB) samples using a QIAamp DNA Blood Mini Kit (Qiagen), taken at diagnosis and/or relapse (range of leukemic blasts: 20% to 100%), and from remission samples. In case no. 10 (**Table 1**) the DNA extracted from BM fibroblasts, was used as germline. DNA was quantified using a Denovix spectrophotometer (Diatech Lab line) according to the manufacturer instructions, and quality was assessed using agarose gel electrophoresis. RNA was extracted from bone marrow or peripheral blood samples using a QIAamp RNA Blood Mini Kit (Qiagen) at a QIAcube automated station. All samples had RNA integrity number ≥6.

Conventional and molecular cytogenetics

Karyotyping and Fluorescence in situ hybridization (FISH). Karyotypes were done after G-banding with Wright stain and described according to the ISCN 2021, on BM/PB

samples. Analysis was carried out on 3-16 metaphases. CI-FISH was performed as previously reported [5]. Eighty cases belonged to a previously published study cohort [5]. Genomic rearrangements of *TERT* were investigated by interphase FISH with a genomic probe spanning the gene (RP11-117B23, UCSC GRCh37/hg19) and with a dual color break apart assay (SPEC TERT Dual Color Break Apart Probe, Zytolight, ZytoVision, Bio Optica, Milan, Italy). The *TRB@::HOXA* rearrangement of cases nos. 5 and 7, was investigated with RP11-1220K2/RP11-556I13 (green) for *TRB@*, and RP5-1103I5/RP1-167F23 (orange) for *HOXA*. To assess the involvement of *ZFP36L2* (case no. 11), we used clones RP11-339H12 (spanning *ZFP36L2,* in orange) and RP11-391M15 (telomeric to *ZFP36L2,* in green) (UCSC GRCh37/hg19), at diagnosis, hematological remission (post-consolidation), and relapse (post-hematopoietic stem cell transplantation). The *STARD4::TCERG1* fusion, in case no.1, was investigated with clones RP11-358F12 (5'*STARD4*), RP11-17K24 (flanking 3'*STARD4*), RP11-105I22 (5'*TCERG1*), and RP11-1006D24 (3'*TCERG1*), in dual color assays (break-apart and single fusion); the deletion of *CSMD1* (case no. 6) was studied with clones RP11-1K11 (5'*CSMD1*, green), and RP11-11P7 (3'*CSMD1*, orange) (UCSC GRCh37/hg19). Additional FISH experiments were done on abnormal metaphases and interphase nuclei applying the Whole Chromosome Paint (WCP) 7 green (MetaSystems s.r.l., Milan, Italy) (case no. 7, **Table 1**), and the LSI *BCL2* break-apart FISH probe (Vysis-Abbott, Milan, Italy) (case no. 8, **Table 1**).

Single nucleotide polymorphism array (SNPa). Copy number alterations (CNAs) and copy-neutral Loss of heterozygosity (cnLOH) were assessed using a High-density CytoScan HD SNP microarray platform (Affymetrix/Thermo Fisher scientific) which contains 2.6 milion of markers, 750k of which are SNPs. Experiments were done using 250 ng of high quality genomic DNA following manufacturer instructions (CytoScan assay user manual, Affymetrix Applied Biosystem Microarray, Thermo Fisher, Milan, Italy). In

cases nos. 6, 10, and 11 (**Table 1**), matched remission BM/PB samples were used for filtering. Protocol was supported by Affymetrix Genechip Command Console (AGCC) software. Results were analyzed using the Chromosome Analysis Suite 4.0 software (Affymetrix Applied Biosystem Microarray, Thermo Fisher, Milan, Italy) and annotated based on GRCh37 (hg19). Filter setting were 200 kb d for CNAs, and 10 Mb, with at least 50 markers, for cnLOH. Cases with matched diagnosis/remission samples (=10) were analyzed without filtering. Polymorphisms were excluded by using publicly available CNV databases (genomic variants (DGV), Online Mendelian Inheritance in Man (OMIM) and University of California Santa Cruz (UCSC). Chromothripsis was defined according to previously established criteria [6–9]. In particular, positive cases had to meet the following criteria: 1) rearrangements confined to one single or very few chromosome(s) or chromosome subregion(s), rather than being scattered throughout the entire genome; 2) copy number profile with changes between two states, namely one or two copies (occasionally three); 3) presence of at least ten changes in segmental copy number between two or three copy number states.

Mutational analysis

Sanger sequencing. Hot spot mutations of *NOTCH1* exons 26, 27, and 34 and of *FBXW7* exons 8 and 9, were investigated in 101/103 cases, while *TERT* promoter (*TERT*p) variants in the 12 T-ALL cases with chromothriptic events (ABI 3500 Genetic analyzer instrument, Applied Biosystems, Monza, Italy). Forward and reverse primers were referred to GRCh37 genomic coordinate system (NM_000005.9, for regulatory core promoter 274 bp) (www.ncbi.nlm.nih.gov/gene, www.ensembl.org/Homo_sapiens) [10,11] Alignment and sequence analyses were supported by Clustal Omega (www.ebi.ac.uk/Tools/msa/clustalo), Ensembl (http://www.ensembl.org/Homo_sapiens), and COSMIC (https://cancer.sanger.ac.uk/cosmic) websites.

Whole Genome Sequencing (WGS). WGS was done by Novogene, (Cambridge, UK) on paired leukemic/germline samples in 5 cases (nos. 3, 6, 7, 10, and 11, **Table 1**) (see **Supplementary Table 3** for UPN). Briefly, the genomic DNA was randomly sheared into short fragments (350 base pairs) which were end repaired, A-tailed, ligated with the full-length adapters of Illumina sequencing, and amplified by PCR. Libraries were checked for size distribution by Agilent 2100 Bioanalyzer (Agilent Technologies, CA, USA), quantified by real-time PCR (to meet the criteria of 3 nM), and sequenced on NovaSeq 6000 (paired end 150bp). Burrows-Wheeler Aligner BWA v 0.7.17[12] was utilized to align the FASTQ to the human reference genome (hg38). BAM files were sorted using SAM tools v1.8[13] and duplicate reads were marked with Picard v2.18.9. Genomic variats were detected with GATK v4.0[14] and annotated with ANNOVAR.[15] Somatic SV and CNV were detected by DELLY Detect v0.7.8 and Control-FREEC v11.4 after filtering with matched germline samples.

Targeted Next generation sequencing (NGS). The Myeloid Solution™ (MYS) was carried out in all 12 cth+ T-ALL cases. It investigated hot-spot regions or the full coding sequence of 30 genes typically involved in myeloid neoplasms, i.e. *ABL1, ASXL1, BRAF, CALR, CBL, CEBPA, CSF3R, DNMT3A, ETV6, EZH2, FLT3, HRAS, IDH1, IDH2, JAK2, KIT, KRAS, MPL, NPM1, NRAS, PTPN11, RUNX1, SETBP1, SF3B1, SRSF2, TET2, TP53, U2AF1, WT1, ZRSR2.* In cases identified by UPN 269, UPN 651, and UPN 282 (see **Supplementary Table 3** for patient UPN) germline samples were used to assess the somatic nature of gene variants. The Hereditary Cancer Solution™ (HCS) was done in the 12 cth+ T-ALL (**Table 1**) and in 10 cth- T-ALL cases (**Supplementary Tables 3 and 4**), to investigate 27 genes implicated in susceptibility to breast and ovarian cancer, hereditary non polyposis colorectal cancer (Lynch syndrome), and intestinal Polyposis Syndromes: *ATM, APC, BARD1, BRCA1, BRCA2, BRIP1, CDH1, CHEK2, EPCAM, FAM175A, MLH1,*

MRE11A, MSH2, MSH6, MUTYH, NBN, PALB2, PIK3CA, PMS2, PSM2CL, PTEN, RAD50, RAD51C, RAD51D, STK11, TP53, and *XRCC2*. Libraries were prepared using 200 ng of BM and PB genomic DNA following manufacturer's instructions. Pooled libraries were sequenced on MiSeq Reagent kit v.3 on Illumina MiSeq Sequencer (Illumina, San Diego, CA). FASTQ files were analyzed with Sophia DDM software™ (version 5.3.9.2). Exonic and splice site variants were taken into consideration and filtered retaining with a global minor allele frequency (MAF) <0.01. The germline nature of the variants was inferred considering those with VAF ≥ 45-60% and ≥ 90% (paying attention to eventual concomitant CNVs), otherwise variants were considered to be somatic. For the interpretation of putative germline variants, we referred to the American College of Medical Genetics and Genomics (ACMG) (7). Somatic variants were classified by mean of FATHMM web server (http://fathmm.biocompute.org.uk) [16]. Benign/likely benign variants were excluded from analysis.

RNA sequencing (RNASeq). RNASeq was performed on 25 T-ALL cases including 6 cth+ (nos. 1, 4, 6, 10-12, **Table 1 and Supplementary Table 3**) and 19 cth- (**Supplementary Table 3**). Libraries were prepared at Novogene, (Cambridge, UK), following the NEBNext® Ultra™ RNA Library Prep Kit on polyA-enriched mRNA, to achieve an average of 50 M reads per sample and were sequenced on Illumina NovaSeq 6000 with 150bp paired-end read run (Illumina San Diego, CA). Low quality reads or reads with adapters were filtered out by using fastp. [17] Cleaned reads were aligned to the human genome (GRCh37/hg19) using STAR and processed with Samtools. [18] Read counts per gene were generated using HTseq package [19] and Bioconductor/R DESeq2 package was used for normalization and differential expression analysis [20] using $padj<0.05$ and log_2FC ≥I2|. Fusion transcripts were identified using STAR-fusion. Functional and integrative analysis of the identified Differentially Expressed Genes (DEGs) was assessed using

WikiPathways on ShinyGO software,[21] Transcription factors target genes analysis was carried out using the ENCODE TF ChIP-seq dataset.[22]

Gene mutations were obtained after ANNOVAR annotation of VCF files. Only exonic and splicing variants with MAF<0.01 according to 1000G, ExAC and ESP650 were retained. Genes whose variants passed all filters were matched with a list of 235 candidate genes involved in DNA repair, telomere biology and T-ALL pathogenesis (**Supplementary Table 17**). Variants found in more than one cth- case were considered non chromothripsis-specific events and were filtered out. The degree of pathogenicity of the filtered variants was assigned by using in silico predictors reported in Varsome database.[23] Only pathogenic/likely pathogenic events were validated by Sanger sequencing.

RNA microarray analysis. The analysis was carried out on 68 T-ALL cases (33 ETP/near ETP, 31 non-ETP, and 4 with unknown stage of blast differentiation). RNA was amplified and hybridized using the “GeneChip® WT PLUS Reagent Kit” (Affymetrix Applied Biosystem Microarray, Thermo Fisher, Milan, Italy) and profiled using the Human Clariom S Assay (Affymetrix Applied Biosystem Microarray, Thermo Fisher, Milan, Italy) which interrogates over 20000 well-annotated genes. The WT PLUS Reagent Kit generates amplified and biotinylated sense-strand DNA targets from total RNA; it uses a reverse transcription priming method that primes the entire length of RNA. Amplification of the cDNA was quantified, fragmented, marked, and prepared for hybridization from the GeneChip® Clariom S Human Array (Thermo Fisher Scientific) for expression level measurement. Hybridization of the microarrays was performed in a GeneChip® Hybridization Oven 645 (Affymetrix/Thermo Fisher Scientific) and after washing and staining, arrays were scanned using the GeneChip Scanner 3000 (Affymetrix Applied Biosystem Microarray, Thermo Fisher, Milan, Italy). Primary data analysis was performed with Transcriptome Analysis Console software version 4.0 (TAC 4.0.2.15). Differentially

expressed RNA were estimated by fold-change filtering combined with Student's t-test. Transcripts with fold change expression ≥ ±2.0 and FDR<0.05 were considered significantly differentially expressed. A supervised hierarchical analysis was performed on 32/33 ETP/near-ETP ALL cases, i.e. 12 *HOXA*, 5 *BCL11B*-a, 2 *TLX3*, and 13 unclassified, with ≥50% of blasts in the DNA sample. They included 11 cth+ and 21 cth- cases.

Supplementary Results

FISH. FISH assays used to investigate abnormalities of the *TERT* gene gave normal hybridization patterns in all cases. In both cases nos. 5 and 7 (**Table 1**) the double color double fusion *TRB@::HOXA* assay confirmed the gene fusion. In addition, in case no. 7, the WCP 7 marked the normal chromosome 7 and the ring chromosome. In case no.8 (**Table 1**) the probe for *BCL2* showed the presence of multiple copies of the gene in two clusters of amplification. In case no. 11 (**Table 1**), the break-apart assay for *ZFP36L2* showed 2 fusions and 1 orange signal in 86% of nuclei, indicating the breakpoint fell within RP11-339H12, the clone that encompasses the gene, thus confirming its involvement in the t(1;2)(p34;p31) translocation. Experiments on remission and at relapse, post hematopoietic stem cell transplantation samples, showed that the *ZFP36L2* rearrangement disappeared at remission (normal hybridization patterns) but re-emerged at relapse (2 fusions and 1 orange signal in 70% of interphase nuclei). In patient no.1, FISH confirmed the occurrence of the *STARD4::TCERG1* fusion (80% of nuclei) with loss of the 3'*STARD4* and the 5'*TCERG1*, as result of a 5q22.1-5q32 deletion*;* in patient no.6 the 3'*CSMD1* partial deletion (1 fusion and 1 green signal in 90% of nuclei).

Targeted Next generation sequencing (NGS). The somatic nature of *ETV6* variants was confirmed in two cases (nos. 6 and 11, **Table 1**), while for *RUNX1* it was assessed in one

case (no.7, **Table 1**). The *ATR* c.2320del, p.I774Yfs*5 (COSM214499) of case 10 (**Table 1**) was germline.

RNASeq. RNAseq identified three gene fusions at chromosome regions involved by chromothriptic events. A rearrangement between exon 4 of *STARD4* and exon 16 of *TCERG1* on chromosome 5 was found in case no 1 (**Supplementary Table 10**). In case no 11, chromothripsis at chromosome 1 produced rearrangements of *SFPQ:* we found an *in frame* transcript between exon 7 of *SFPQ* and exon 2 of *ZFP36L2,* i.e. the *SFPQ::ZFP36L2*, which likely produced a 1084 amino acid fusion protein, and an *out-of-frame* fusion involving exon 9 of *SFPQ* and exon 3 of *PLEKHG4B/CTD-2231H16.1*, i.e. the *SFPQ::PLEKHG4B/CTD-2231H16.1*, that generated a stop codon on *PLEKHG4B*, at amino acid 24 (**Supplementary Table 10**). The *MYCN*P44L was not detected in any case. Expression analysis identified 111 DEGs, i.e. 50 down- and 61 up-regulated, that differentiated cth+ (n. 6) *vs* cth- (n.19) cases (**Supplementary Fig. 2a, Supplementary Table 12**). The functional annotation of all DEGs, showed that the downregulation of nitric oxide related genes, i.e. *AOX1* and *HBA1*, and the upregulation of the *NOTCH* signaling (*MFAP2*, *JAG2* and *DLK1* genes) were the top enriched pathways in cth+ (FDR < 0.1) (**Supplementary Fig. 2b**). Moreover, 30 out of the 111 DEGs (27%), were targets of *EZH2* (FDR < 0.1) (**Supplementary Fig. 2c**), 24 of which were upregulated (**Supplementary Table 12**). RNA-Seq confirmed the downregulation of *RGCC.*

RNA microarray analysis. Supervised hierarchical analysis distinguished 4 subgroups: *HOXA* (=13 cases), i.e. 12 cases with *HOXA*-related abnormalities and case CTH11+ (no.11 of **Table 1**) with the t(1;2)(p34;p21)/*SFPQ::ZFP36L2*; *BCL11B*-a (=5); *TLX3* (=3), i.e. 2 cases with *BCL11B*::*TLX3* and 1 case undetermined; and the subgroup of unclassified cases (=11). Confirming these results, the CTH11+ case clustered with *HOXA*s in a supervised analysis of *HOXA* positive T-ALL *vs* all other cases. The *HOXA*

signature was characterized by 159 differentially expressed genes, namely 124 down-regulated and 35 up-regulated. Among the upregulated genes there were *MEIS*, *HOXA9-HOXA10*, and *HOXA13*. Case CTH11+ had high levels of *HOXA13* expression (**Supplementary Figure 3**).

Whole Genome sequencing. All somatic SV and CNV detected in 5 cth+ T-ALL cases are summarized in **Supplementary Table 6**. All chromothriptic events detected by SNPa were confirmed by WGS and additional catastrophic events were unraveled. The results of exonic variants analysis of 235 candidate genes, listed in **Supplementary Table 17,** are shown in **Supplementary Table 18**. All somatic SNV/Indel detected in the 5 cases cth+ T-ALL are reported in **Supplementary Table 21**.

References

1 Coustan-Smith E, Mullighan CG, Onciu M, Behm FG, Raimondi SC, Pei D *et al.* Early T-cell precursor leukaemia: a subtype of very high-risk acute lymphoblastic leukaemia. *Lancet Oncol* 2009; **10**: 147–156.

2 Liu Y, Easton J, Shao Y, Maciaszek J, Wang Z, Wilkinson MR *et al.* The genomic landscape of pediatric and young adult T-lineage acute lymphoblastic leukemia. *Nat Genet* 2017; **49**: 1211–1218.

3 Morita K, Jain N, Kantarjian H, Takahashi K, Fang H, Konopleva M *et al.* Outcome of T-cell acute lymphoblastic leukemia/lymphoma: Focus on near-ETP phenotype and differential impact of nelarabine. *Am J Hematol* 2021; **96**: 589–598.

4 Bardelli V, Arniani S, Pierini V, Di Giacomo D, Pierini T, Gorello P *et al.* T-cell acute lymphoblastic leukemia: Biomarkers and their clinical usefulness. Genes (Basel). 2021; **12**. doi:10.3390/genes12081118.

5 La Starza R, Pierini V, Pierini T, Nofrini V, Matteucci C, Arniani S *et al.* Design of a Comprehensive Fluorescence in Situ Hybridization Assay for Genetic Classification of T-Cell Acute Lymphoblastic Leukemia. *J Mol Diagnostics* 2020; **22**: 629–639.

6 Stephens PJ, Greenman CD, Fu B, Yang F, Bignell GR, Mudie LJ *et al.* Massive genomic rearrangement acquired in a single catastrophic event during cancer development. *Cell* 2011; **144**: 27–40.

7 Rausch T, Jones DTW, Zapatka M, Stütz AM, Zichner T, Weischenfeldt J *et al.* Genome sequencing of pediatric medulloblastoma links catastrophic DNA rearrangements with TP53 mutations. *Cell* 2012; **148**: 59–71.

8 Korbel JO, Campbell PJ. Criteria for inference of chromothripsis in cancer genomes. Cell. 2013; **152**: 1226–1236.

9 Rode A, Maass KK, Willmund KV, Lichter P, Ernst A. Chromothripsis in cancer cells: An update. Int. J. Cancer. 2016; **138**: 2322–2333.

10 Nofrini V, Matteucci C, Pellanera F, Gorello P, Di Giacomo D, Lema Fernandez AG *et al.* Activating somatic and germline TERT promoter variants in myeloid malignancies. *Leukemia* 2021; **35**: 274–278.

11 Pierini T, Nardelli C, Lema Fernandez AG, Pierini V, Pellanera F, Nofrini V *et al.* New somatic TERT promoter variants enhance the Telomerase activity in Glioblastoma. *Acta Neuropathol Commun* 2020; **8**. doi:10.1186/s40478-020-01022-4.

12 Li H, Durbin R. Fast and accurate long-read alignment with Burrows-Wheeler transform. *Bioinformatics* 2010; **26**. doi:10.1093/bioinformatics/btp698.

13 Li H, Handsaker B, Wysoker A, Fennell T, Ruan J, Homer N *et al.* The Sequence

Alignment / Map (SAM) Format and SAMtools 1000 Genome Project Data Processing Subgroup. *Bioinformatics* 2009; **25**.

14 McKenna A, Hanna M, Banks E, Sivachenko A, Cibulskis K, Kernytsky A *et al.* The genome analysis toolkit: A MapReduce framework for analyzing next-generation DNA sequencing data. *Genome Res* 2010; **20**. doi:10.1101/gr.107524.110.

15 Wang K, Li M, Hakonarson H. ANNOVAR: Functional annotation of genetic variants from high-throughput sequencing data. *Nucleic Acids Res* 2010; **38**. doi:10.1093/nar/gkq603.

16 Shihab HA, Gough J, Cooper DN, Day INM, Gaunt TR. Predicting the functional consequences of cancer-associated amino acid substitutions. *Bioinformatics* 2013; **29**: 1504–1510.

17 Chen S, Zhou Y, Chen Y, Gu J. Fastp: An ultra-fast all-in-one FASTQ preprocessor. In: *Bioinformatics*. 2018, pp i884–i890.

18 Dobin A, Davis CA, Schlesinger F, Drenkow J, Zaleski C, Jha S *et al.* STAR: Ultrafast universal RNA-seq aligner. *Bioinformatics* 2013; **29**: 15–21.

19 Anders S, Pyl PT, Huber W. HTSeq-A Python framework to work with high-throughput sequencing data. *Bioinformatics* 2015; **31**: 166–169.

20 Love MI, Huber W, Anders S. Moderated estimation of fold change and dispersion for RNA-seq data with DESeq2. *Genome Biol* 2014; **15**. doi:10.1186/s13059-014-0550-8.

21 Ge SX, Jung D, Jung D, Yao R. ShinyGO: A graphical gene-set enrichment tool for animals and plants. *Bioinformatics* 2020; **36**. doi:10.1093/bioinformatics/btz931.

22 ENCODE Project Consortium T, coordination O, production leads D, analysts L,

group W, project management N *et al.* An integrated encyclopedia of DNA elements in the human genome ENCODE Encyclopedia of DNA Elements. *Nature* 2012; **489**.

23 Kopanos C, Tsiolkas V, Kouris A, Chapple CE, Albarca Aguilera M, Meyer R *et al.* VarSome: the human genomic variant search engine. *Bioinformatics* 2019; **35**: 1978–1980.

Supplementary Fig. 1. Oncosuppressor genes that underwent monoallelic deletion due to chromothriptic events

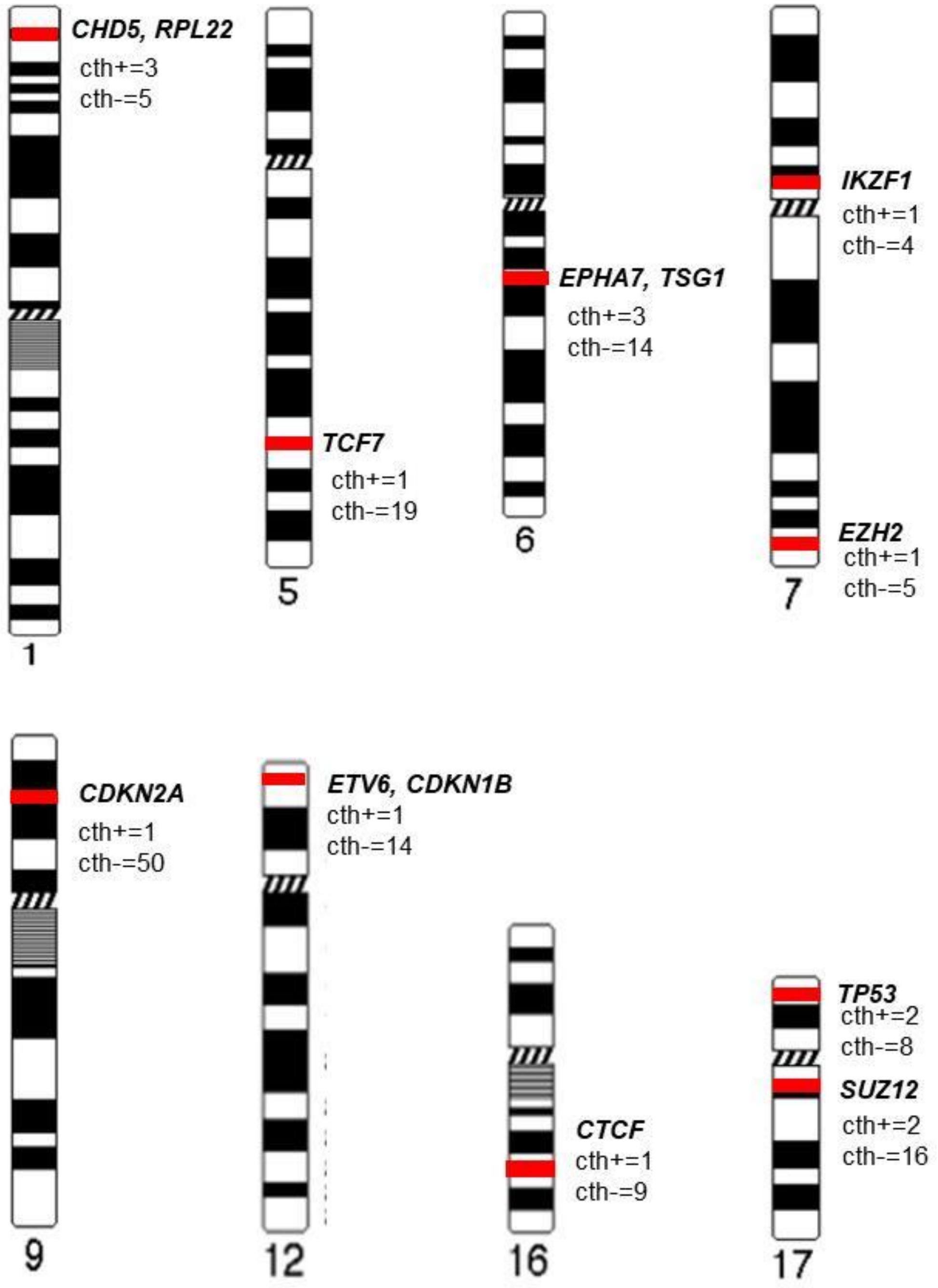

Legend. Genes undergoing deletions in cth+ and cth- T-ALL cases, the chromosomal band where they are located, and the number of cases affected, were indicated on ideograms; cth, chromothripsis.

Supplementary Fig. 2. RNA-Seq gene expression analysis of 6 cth+ versus 19 cth- T-ALL cases

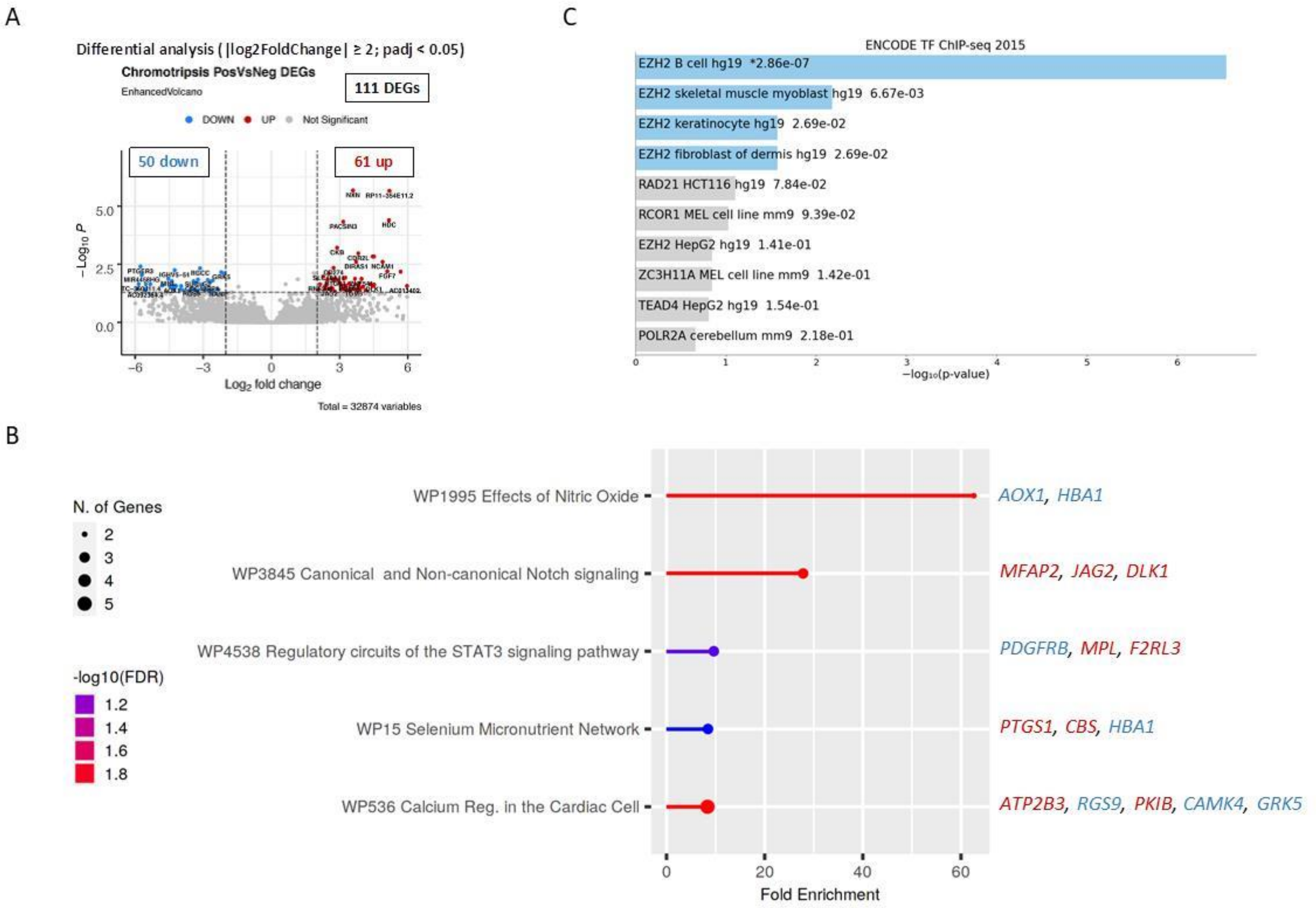

Legend. Summary of RNA-Seq gene expression analysis of cth+ versus cth-. **A)** Volcano plot showing the distribution of the 111 identified DEGs (padj < 0.05) in up- (log2 Fold Change > 2) or down-regulated (log2 Fold Change < -2). **B)** Schematic representation of significantly enriched pathways (FDR < 0.1), including the respective genes (red: up-, blue: down-regulated). Functional analysis of the 111 DEGs was performed using WikiPathways on ShinyGO software. C) Bar plot showing the transcription factors acting on the 111 DEGs using the ENCODE TF ChIP-seq dataset on Enrichr (* FDR < 0.1).

Supplementary Fig. 3. Supervised hierarchical clustering of ETP/near-ETP ALL cases (panels A-C):

A) Clustering of cases based on underlying genomic rearrangements confirmed 4 subgroups, i.e. *HOXA*, *BCL11*B-a, *TLX3*, and unclassified. Unclassified CTH+11 (case no.11 of Table 1), harboring *SFPQ::ZFP36L2,* grouped with *HOXA*-positive ETP/near-ETP ALL (black arrow)

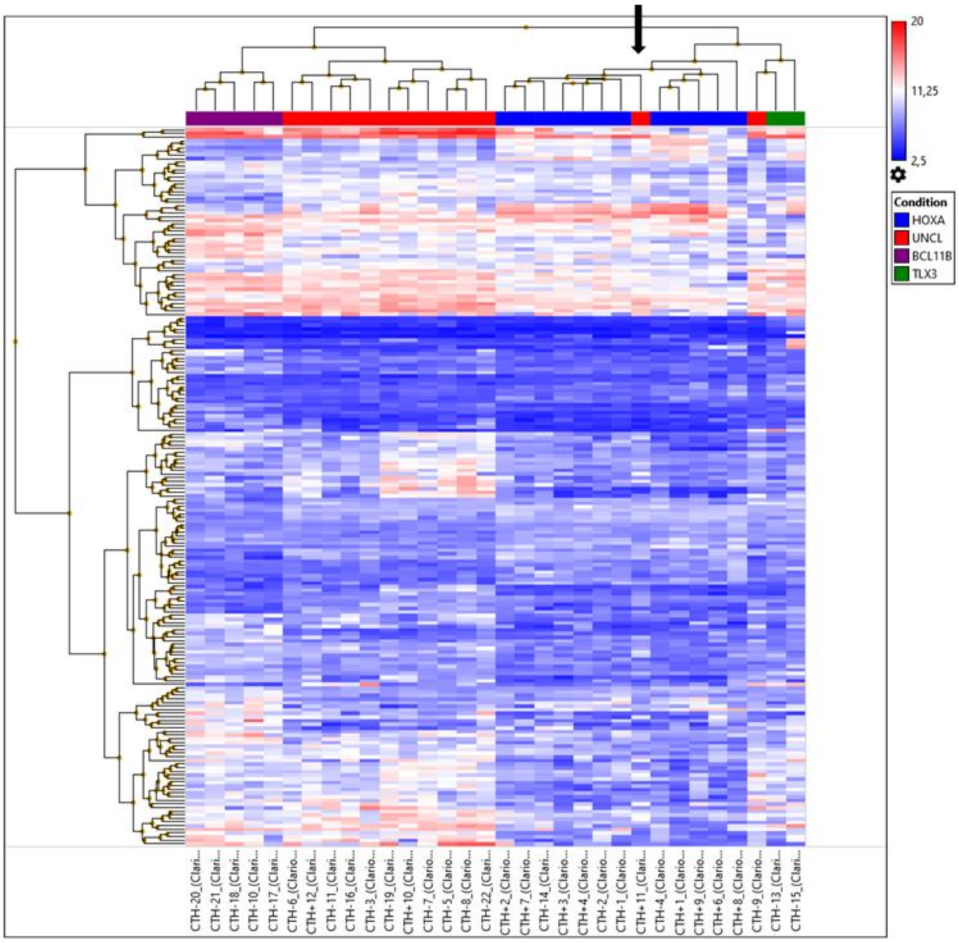

B) Supervised analysis of *HOXA*-positive cases *vs* all the other subtypes confirmed that case CTH11+ belonged to the *HOXA* category (black arrow)

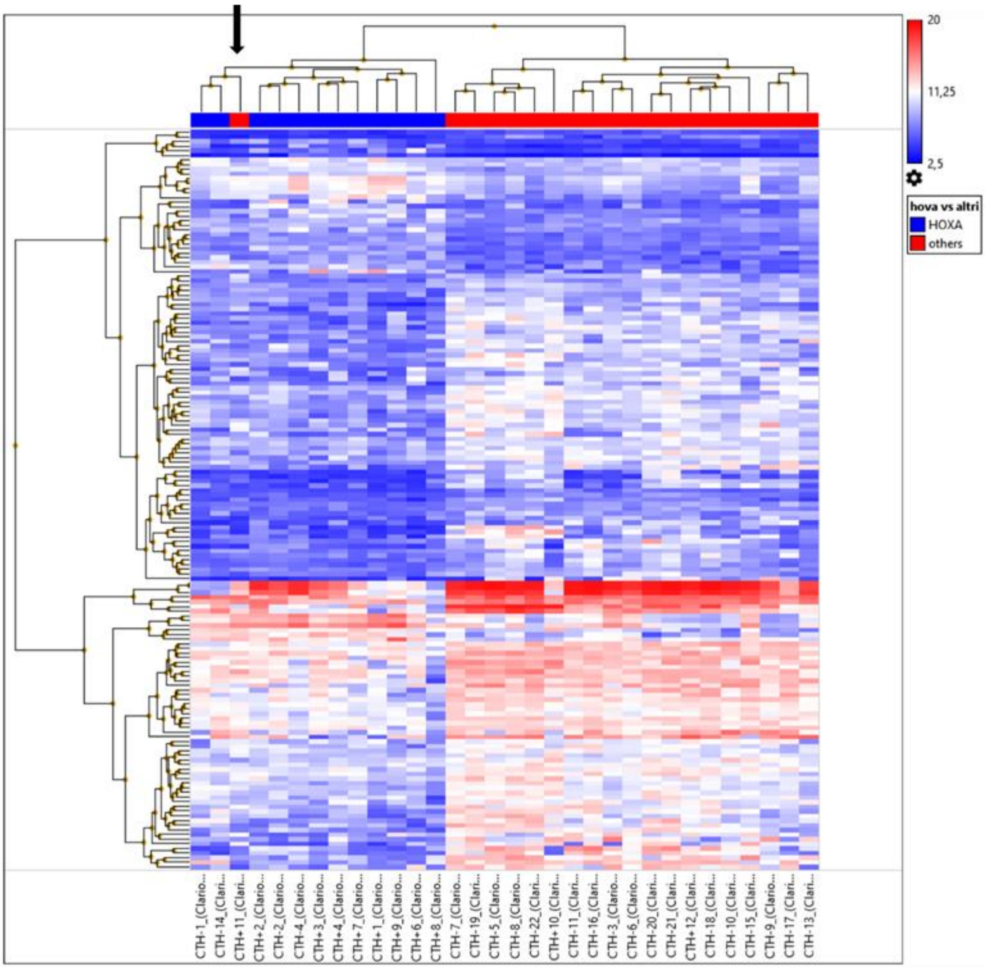

C) The levels of *HOXA13* expression was significantly higher in *HOXA*-positive ETP/near-ETP ALL (12 cases with genomic abnormalities related to HOXA upregulation) than in the other subgroups (=20) (Mann Whitney Test; p=0.002). Red square corresponds to case CTH11+ (no.1 of Table1)

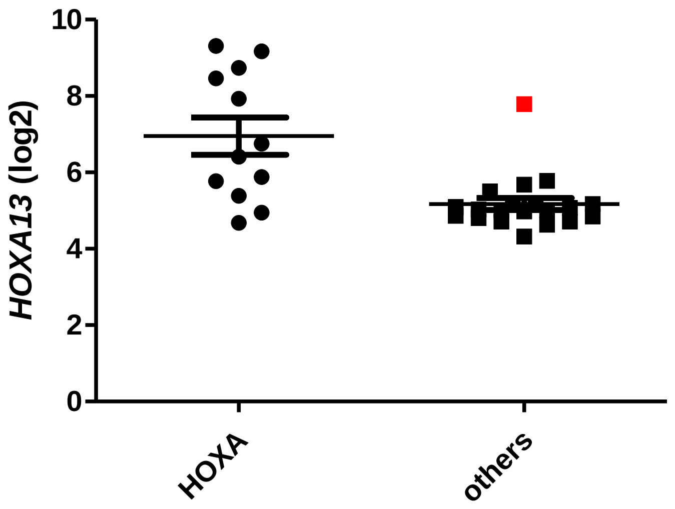

Supplement: Supplementary file 1 — Supplementary Information [file 41375_2022_1671_MOESM1_ESM.pdf]
